# Nanoscale All-Oxide-Heterostructured Bio-inspired Optoresponsive Nociceptor

**DOI:** 10.1007/s40820-020-00419-z

**Published:** 2020-04-01

**Authors:** Mohammad Karbalaei Akbari, Jie Hu, Francis Verpoort, Hongliang Lu, Serge Zhuiykov

**Affiliations:** 1 Centre for Environmental and Energy Research, Ghent University Global Campus, Incheon, South Korea; 2 Department of Green Chemistry and Technology, Faculty of Bioscience Engineering, Ghent University, 9000 Ghent, Belgium; 3 College of Information Engineering, Taiyuan University of Technology, Taiyuan, 030024 Shanxi People’s Republic of China; 4 Laboratory of Organometallics, Catalysis and Ordered Materials, State Key Laboratory of Advanced Technology for Materials Synthesis and Processing, Wuhan University of Technology, Wuhan, 430070 People’s Republic of China; 5 School of Microelectronic, Fudan University, Shanghai, 200433 People’s Republic of China

**Keywords:** 2D heterostructures, Artificial nociceptors, Bio-inspired device, Heterointerfaces engineering

## Abstract

Retina nociceptor, as a key sensory receptor, not only enables the transport of warning signals to the human central nervous system upon its exposure to noxious stimuli, but also triggers the motor response that minimizes potential sensitization. In this study, the capability of two-dimensional all-oxide-heterostructured artificial nociceptor as a single device with tunable properties was confirmed. Newly designed nociceptors utilize ultra-thin sub-stoichiometric TiO_2_–Ga_2_O_3_ heterostructures, where the thermally annealed Ga_2_O_3_ films play the role of charge transfer controlling component. It is discovered that the phase transformation in Ga_2_O_3_ is accompanied by substantial jump in conductivity, induced by thermally assisted internal redox reaction of Ga_2_O_3_ nanostructure during annealing. It is also experimentally confirmed that the charge transfer in all-oxide heterostructures can be tuned and controlled by the heterointerfaces manipulation. Results demonstrate that the engineering of heterointerfaces of two-dimensional (2D) films enables the fabrication of either high-sensitive TiO_2_–Ga_2_O_3_ (Ar) or high-threshold TiO_2_–Ga_2_O_3_ (N_2_) nociceptors. The hypersensitive nociceptor mimics the functionalities of corneal nociceptors of human eye, whereas the delayed reaction of nociceptor is similar to high-threshold nociceptive characteristics of human sensory system. The long-term stability of 2D nociceptors demonstrates the capability of heterointerfaces engineering for effective control of charge transfer at 2D heterostructured devices.

**Electronic supplementary material:**

The online version of this article (10.1007/s40820-020-00419-z) contains supplementary material, which is available to authorized users.

## Highlights

- Artificial optoelectronic nociceptor based on two-dimensional heterostructured all-oxide nanostructures was designed.
- Two-dimensional heterointerfaces were functionalized, and their engineering toward fabrication of artificial nociceptors was confirmed.

**Electronic supplementary material:**

The online version of this article (10.1007/s40820-020-00419-z) contains supplementary material, which is available to authorized users.

## Graphical abstract

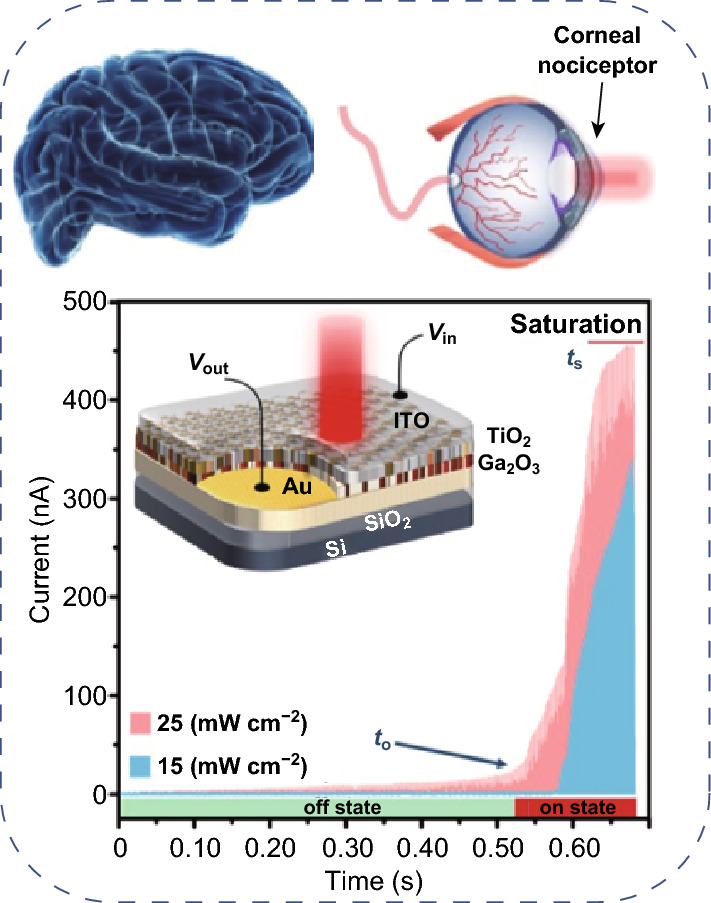

## Introduction

**Fig. 1:**
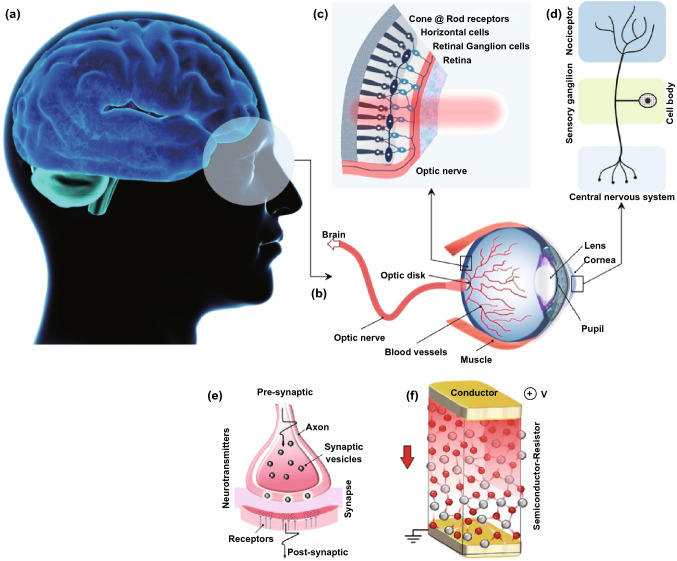
The scheme of human eye receptor and nociceptor system. **a** Human brain as decision making unit receives the informative signals from **b** human eye and its sensory components including the **c** light receptors and nociceptors in retina section. **d** Typical nociceptor with its components. **e** Typical schematic representation of a natural synapse and **f** its artificial counterparts in conductor/semiconductor/conductor-sandwiched configuration

Mimicking the human brain functionalities by using neuromorphic-based technologies is a quite essential achievement toward the development of the artificially engineered bio-inspired electronic devices [1]. The emulation of human sensory system and the sensorimotor functionalities are significant hurdles in biomimetic studies. These challenges inevitably should be responded as the substantial step toward the creation of artificial bio-inspired systems [2]. The visual processing is one of the fundamental and prominent functionalities of the human brain (Fig. 1a). Visual processing is fulfilled by outstanding features of the human’s eye [3]. Eye, as the natural visual detector and processor (Fig. 1b), consists of a large number of receptors (Fig. 1c) and nociceptors (Fig. 1d). In fact, nociceptor is a key sensory receptor that recognizes noxious stimuli, which in turn generates and delivers the warning signals to the central nervous system. The brain and nervous system then generate commands to trigger the motor responses and then to minimize the potential sensitization [4]. Visual cognition is fulfilled after the processing of directly captured and detected optical stimuli by the eye’s cone and rod receptors in retina [5]. Then, the generated bio-chemical voltaic signals are transferred by retinal ganglion cells to the optical nerve and finally are transmitted into the visual cortex of the brain for further processing [5].

The cornea has the highest number of nociceptors in the human eye, and the majority of the corneal nociceptors are polymodal [6]. The polymodal nociceptors are activated by the mechanical, heat, and cold stimuli and also by a large variety of exogenous irritant chemicals and endogenous inflammatory substances which are released by the damaged corneal tissues [7]. The noxious stimulations elicit the potential signals in the sensory endings of nociceptors, which are transferred and conducted by the optical nerve axons to the brain stem, and consequently evoke the nociceptive pain. Noteworthy, nociceptors play their role in two individual modes, i.e., normal and abnormal states [6]. The “threshold” and “relaxations” are two main characteristics of nociceptors in the normal state [7]. The nociceptor is in off-mode when the intensity of stimulus is lower than the threshold value, whereas the same nociceptor is turned on and reacts strongly once the stimulus intensity exceeds the threshold value. The relaxation time is another nociceptor’s characteristic in the normal state, which refers to the required time span for the ultimate retrieval of nociceptor to the off-state [6, 7]. Within the relaxation time span, the required intensity of stimulus for re-ignition of nociceptors is lower than the threshold values, since nociceptors are already activated. The abnormal state deals with the condition when the nociceptor is damaged. It happens once the nociceptor experiences stronger stimulus signals than its threshold limit. In this state, the nociceptor basically performs similar to the human receptor [8]. In the abnormal mode, *allodynia* refers to the condition that nociceptor responses to the signals even under threshold values and *hyperalgesia* is the condition in which the reaction to the provoking signals is stronger than that of in the normal state [8]. Typical examples of abnormal nociceptive responses are the blinking reaction of the damaged eye to the daylight or the unexpected response of the burned skin to the normal heat sources. Looking at the nervous system, the biological synapse is the fundamental base of sensorimotor system facilitating various functionalities including the pain signal transfer in the neural system (Fig. 1e). The analogous artificial nociceptive device with similar synaptic functionalities is composed of a semiconductor ultra-thin film sandwiched between two conductive layers (Fig. 1f).

In this study, we report for the first time optical artificial nociceptors built upon ultra-thin amorphous all-oxide heterostructures with ionic transport, anisotropic electrical characteristics, and ultimate transparency. Fabricated nociceptor represents the electronic memristor [9] with both insulating and semimetallic characteristics and controllable charge transfer. These capabilities in turn could facilitate the fabrication of electronic devices whose resistive functions are controlled by their nano-structural modification [10]. In this concept, sub-stoichiometric amorphous gallium oxide (Ga_2_O_3_) thin films (~ 5.0 nm) were rapidly thermally annealed (RTA) in either Ar or N_2_ atmosphere with subsequent quenching. Then, RTA gallium oxide films were utilized as the components of transparent TiO_2_–Ga_2_O_3_ optical memristor in heterostructured configuration. Due to the considerable electron affinity difference between the Ga_2_O_3_ (4.3 eV) and TiO_2_ (1.59 eV) films, the internal driving force can facilitate the charge transfer from the TiO_2_ layer to the adjacent Ga_2_O_3_ neighbor. A charge trapping/de-trapping-associated phenomenon was characterized during the resistive switching (RS) performance of all-oxide-heterostructured devices. Phase reconfiguration in the Ga_2_O_3_ thin film was accompanied by the substantial jump in its conductivity induced by an internal redox reaction of amorphous structure. The distinguished charge transfer mechanism in our devices allows the reproduction of the critical nociceptive characteristics in the optically ignited heterostructured memristor. All vital nociceptor functions, including the no adaptation and sensitization, have been demonstrated in the single instrument at the same time. Furthermore, the experimental manipulation of heterointerfaces facilitated the development of either high-sensitive TiO_2_–Ga_2_O_3_ (Ar) or high-threshold TiO_2_–Ga_2_O_3_ (N_2_) artificial nociceptors. The artificial nociceptive sensors could have several undeniable applications for conditional detection of UV- and γ-radiation in hazardous environment such as aerospace technologies.

## Experimental

### Fabrication and Characterization of Nociceptors

The Au electrodes were patterned by electron beam (EB) evaporation on the SiO_2_/Si substrate. Plasma-enhanced atomic layer deposition (PE-ALD) was employed to deposit 5.0-nm-thick gallium oxide films over Au electrodes. The tris (2,2,6,6-tetramethyl-3,5-heptanedionato) gallium (III), [Ga(TMHD)_3_] (Strem Chemicals, 99%), and O_2_ plasma were used in PE-ALD process. For RTA treatment, the Au–Ga_2_O_3_ electrodes were thermally annealed in controlled condition under Ar or N_2_ atmosphere. The heating rate was designed to be 60 °C min^−1^. The samples were hold for 10 min at the designed temperatures and then finally quenched to the room temperature by the same rate. The 20.0-nm-thick amorphous TiO_2_ films were then deposited by PE-ALD (tetrakis dimethylamino titanium and O_2_ plasma) over the RTA gallium oxide films to develop all-oxide heterostructures. At the final stage, Pt or indium tin oxide (ITO) top electrodes were fabricated over all-oxide-heterostructured films.

### Materials Characterization

Various characterization techniques were employed for investigation of the material properties. The Raman measurements were used individually by continuous laser beam of Raman (*λ *= 750 nm, HORIBA micro-Raman, Lab Ram ARAMIS) to characterize ultra-thin films. To investigate the memristive behavior of heterostructured films, Raman measurements were individually performed at the zero bias and then at the HRS mode of memristor device. XPS studies were extensively used to evaluate the chemical compositions, percentage of elements, and vacancies in the RTA and heterostructured films as well as to determine the band alignment at semiconductor heterointerfaces (XPS, Thermo Scientific theta probe). The absorbance and reflectance spectra of samples were measured using UV–visible diffused reflectance spectrometer (Shimadzu, UV–Vis 2600) to evaluate the bandgap of heterostructured films. To this aim, all films were deposited on the highly transparent glass substrates. The field emission scanning electron microscope (FESEM JEOL-7800F) was used for measurement of photoluminescence characteristics. Hall-effect measurements (Ecopia) were employed to measure the conductivity of the samples. Kelvin probe force microscopy was used to measure the surface potential of samples.

### Memristor and Nociceptor Devices

Autolab Metrohm (PGSTAT204) instrument was used to evaluate the performance of memristors. Tunable LED laser driver (*λ *= 360 nm) in combination with the Autolab signal analyzer (PGSTAT204) was employed to measure the photoresponsive and nociceptive responses of devices and to precisely design and pattern optical pulses.

## Results and Discussion

### Structural Properties

**Fig. 2:**
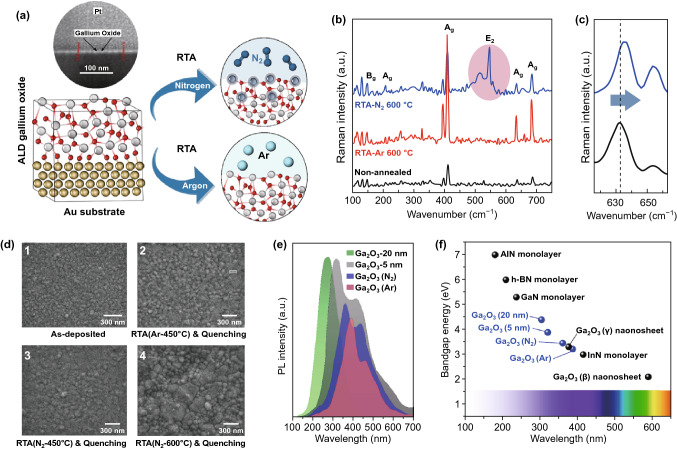
Schematic of RTA treatment accompanied by characterization results of ultra-thin heterostructured films. **a** Graphical scheme for RTA of 5-nm-thick gallium oxide film in Ar and N_2_ atmosphere. **b** Typical Raman characteristics of RTA-treated gallium oxide films in Ar and N_2_ atmosphere, **c** Raman shifts and **d** FESEM microstructural observations. **e** Photoluminescence spectra of as-deposited and RTA-treated gallium oxide films. **f** Comparative diagram of *E*_g_ of selected two-dimensional (2D) metal nitrides accompanied by the results of present study

It was observed that the RTA of Ga_2_O_3_ film in Ar and N_2_ atmospheres (Fig. 2a) and the subsequent quenching were accompanied by the phase transformation [10]. The Raman spectra (Fig. 2b) of the samples show the characteristic peaks of GaO bonding. Specifically, the observed low-frequency modes of 208 cm^−1^ (A_g_), 170 cm^−1^ (A_g_), and 145 cm^−1^ (B_g_) are the characteristic vibration modes for liberation and vibration of tetrahedral–octahedra chain in β-Ga_2_O_3_ [11]. The other observed peak at 415 cm^−1^ is assigned to the in-plane octahedra-related optical mode of Ga_2_O_6_ [11]. The A_g_ characteristic peaks at 634 and 679 cm^−1^ are attributed to the tetrahedra-related optical modes of GaO_4_, respectively [11]. The intensity of characteristic modes of GaO bonding increased after RTA. The characteristic E_2_ (high) Raman mode of GaN was also detected at 548 cm^−1^ in Ga_2_O_3_ annealed in N_2_ atmosphere confirming the successful nitrogen incorporation in Ga_2_O_3_ [12]. The RTA process of as-deposited Ga_2_O_3_ in N_2_ atmosphere was accompanied by the red shift of A_g_ characteristic peaks of Ga_2_O_3_, which can be attributed to the crystalline state of RTA Ga_2_O_3_ film. From the morphological point of view, the RTA resulted in the visible growth of Ga_2_O_3_ grains (Fig. 2d1–d4). One of the most notable observations in our study is the prominent alteration of bandgap energy (*E*_g_) of Ga_2_O_3_ thin films. The photoluminescence (PL) measurements (Fig. 2e) accompanied by calculated bandgap (Fig. 2f) confirmed the decrease of *E*_g_ from 4.47 to 3.89 eV, as the thickness of Ga_2_O_3_ was reduced from ~ 20.0 to ~ 5.0 nm (Fig. S1a). It can be attributed to the effect of strain-related factors on *E*_g_ of ultra-thin films [12] and to the stoichiometric changes of Ga_2_O_3_ films [13]. The *E*_g_ values of RTA-treated Ga_2_O_3_ films in Ar (3.22 eV) and N_2_ (3.39 eV) atmosphere are smaller than *E*_g_ of as-deposited Ga_2_O_3_ films (3.89 eV) (Figs. 2e, f and S1b, c). Furthermore, the other absorption tails were detected in PL spectra of as-deposited and RTA Ga_2_O_3_ films at wavelengths of 400 ~ 500 nm. It is expected that the visible light PL responsivity is attributed to the extra states in the bandgap of Ga_2_O_3_ caused by the gallium excess in sub-stoichiometric films [10, 14]. It seems that the UV categorized PL peaks in ultra-thin Ga_2_O_3_ films are also related to the crystallization of β-Ga_2_O_3_ in amorphous films (bandgap 3.89 eV). This eventually leads to the dual bandgap properties, which is typical characteristic of the composite materials (Fig. S1). The plot in Fig. 2f represents a comparative scheme of the *E*_g_ values of various ultra-thin 2D nitrides [11, 15]. Graph demonstrates that the RTA-treated and quenched ultra-thin Ga_2_O_3_ samples can be considered as candidates for the tunable optoelectronic applications. The bandgap of the as-deposited Ga_2_O_3_ ultra-thin film decreased after RTA in N_2_ atmosphere. The observation of absorption edge in transmittance spectra of N_2_-doped Ga_2_O_3_ film (Fig. S2) suggests the dissociation of nitrogen anions into Ga_2_O_3_ structure. This implies that the introduction of ionic nitrogen into Ga_2_O_3_ considerably altered its band structure. The origin of the decreased bandgap was attributed to the N acceptor states in the bandgap of Ga_2_O_3_ [16–18]. The other evidences about bandgap alteration of nitrogen-doped ultra-thin Ga_2_O_3_ film are discussed in the next section.

### Mechanism of Phase Transition

**Fig. 3:**
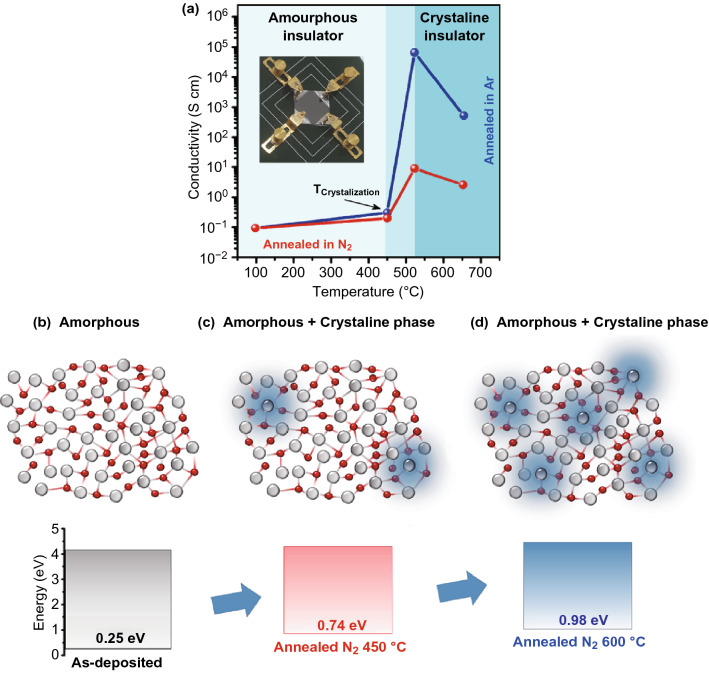
The results of electrical conductivity measurements and graphical scheme of phase transformation of gallium oxide film during RTA treatment and their XPS measurements. **a** Conductivity variation of gallium oxide thin films after RTA in Ar and N_2_ atmosphere. The conductivity was measured after quenching of samples from high temperatures. **b**–**d** The phase transformation in gallium oxide films during RTA treatment. The as-deposited films are amorphous until the crystallization temperature at which the nucleation of crystalline stoichiometric Ga_2_O_3_ nucleus sites occurs [10]. It is accompanied by the increase in Ga excess in remained amorphous phase and the evolution of conductivity of thin films

Figure 3a shows the results of conductivity measurements versus the RTA processing temperature. All measurements were performed after rapid quenching to investigate the final structural phases of materials. It was observed that as-deposited Ga_2_O_3_ samples are in insulating mode, whereas for the samples annealed at 450 °C, the conductivity is still low (~ 10^−1^ S cm^−1^) and transparency is as high as 84% in the visible region (Fig. S2). Noteworthy, measurements confirmed a profound increment of conductivity (Fig. 3a) at the RTA temperatures above 525 °C, where the conductivity (RTA in Ar) is about six orders of magnitude higher than that of the as-deposited samples. The observed jump in conductivity value is incredible. However, further annealing at higher temperature is accompanied by the conductivity decline. These facts reinforce the hypothesis that our ultra-thin films have experienced the phase transformation from *insulator* to *semiconductor* and then to *semimetallic* structures after RTA and fast quenching treatments [10]. A model has been introduced which relates the conductivity increment phenomenon to the nucleation of crystalline and stoichiometric Ga_2_O_3_ nucleus in amorphous gallium oxide [10] (Fig. 3b–d). It is suggested that further nucleation of stoichiometric Ga_2_O_3_ phases is accompanied by the increase in metallic content in ultra-thin films owing to the initial Ga excess in the sub-stoichiometric microstructure. It is expected that the Ga interstitials enhance the number of donors in amorphous Ga_2_O_3_. Therefore, the remained amorphous Ga_2_O_3_ in RTA sample is heavily donor-doped semiconductor. It was suggested that Ga excess increases the possibility of formation of new electronic states above the valence state of amorphous Ga_2_O_3_ [10]. It is confirmed that the number of states in the optical bandgap of amorphous Ga_2_O_3_ increases by enhancement of Ga content in microstructure. It consequently results in the decrease in energy gap between the highest occupied states and the conduction band [10, 19]. Our observations also confirmed the bandgap decrease (Fig. S1) and alteration of the valence band maximum (VBM) of the annealed samples in Ar atmosphere (Fig. S3).

**Fig. 4:**
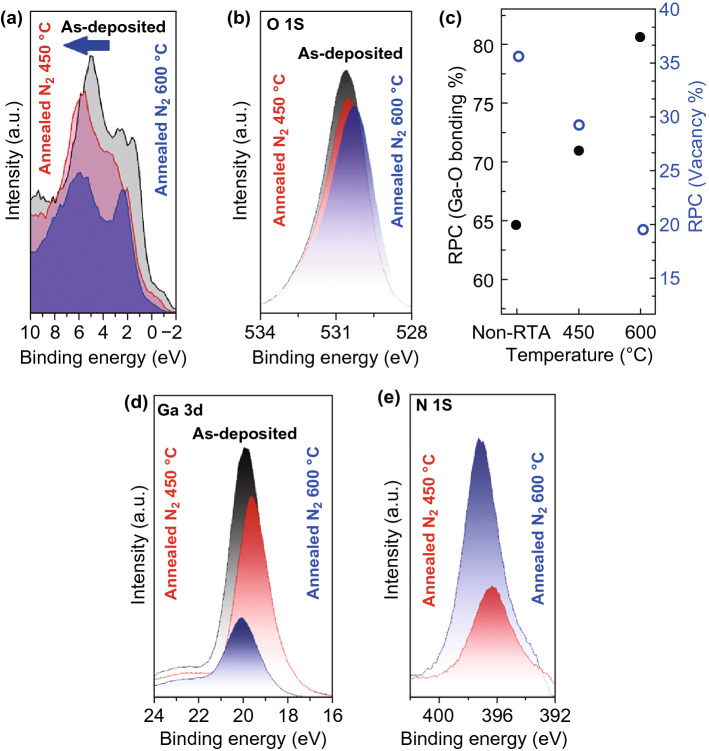
XPS core-level spectra and VBM of N_2_-doped Ga_2_O_3_ films. **a** RTA process is resulted in the alteration of bandgap alignment in gallium oxide films where a higher RTA temperature in N_2_ atmosphere was accompanied by the increase in VBM. **b** Analytical analysis of O1s XPS characteristics of samples, **c** variation of percentage of Ga–O bonding and oxygen vacancy in RTA samples. **d** XPS characteristics of Ga3d and **e** N1s in RTA samples

In the case of RTA of Ga_2_O_3_ film in N_2_, Raman measurements depicted strong characteristic peak of GaN (Fig. 2b). The conductivity jump in RTA (N_2_) Ga_2_O_3_ sample is only two times higher than the conductivity increase in as-deposited Ga_2_O_3_ film, which is much smaller than that of sample annealed in Ar atmosphere (Fig. 3a). Typical chemical composition in the stoichiometric Ga_2_O_3_ is Ga:O = 0.40:0.60. However, the chemical composition of as-deposited sample was 0.46:0.54. Using XPS results, the composition of the RTA (N_2_) samples at 450 °C is obtained as Ga:O:N = 0.59:0.35:0.06, which also demonstrates the Ga excess in the annealed structures. It is expected that the nitrogen is substituted for oxygen atoms in the Ga_2_O_3_ structure and plays its role as the acceptor atom with subsequent partial compensation of the donor effects [10, 16]. Owning to this partial donor compensation, the bandgap of RTA (N_2_) samples is larger than that of RTA (Ar) samples (Fig. 2f). Nevertheless, the bandgap is decreased and the visible light responsivity is facilitated due to the Ga excess in the remained amorphous phase [10]. On the contrary, typical composition of the samples annealed (N_2_-600 °C) is Ga:O:N = 0.67:0.24:0.09, which displays higher level of Ga excess and N_2_ doping in ultra-thin films. The most plausible explanation for higher Ga excess can be related to higher number of crystalline nucleus sites at higher RTA temperature. This phenomenon consequently resulted in the increase in Ga excess in ultra-thin Ga_2_O_3_ film. After annealing in N_2_ atmosphere, the VBM was increased to higher binding energies, as it is graphically depicted (Fig. 3b–d). The XPS results in Fig. 4a show the increase in VBM to higher binding energies after annealing in N_2_ atmosphere at higher annealing temperature. It can be related to higher concentration of N_2_ acceptor in the structure. The O1s core-level spectra of N_2_-annealed samples (Fig. 4b) shifted to lower binding energies. It is because of replacement of N_2_ atoms (with low electronegativity) with O_2_ atoms (with high electronegativity) in Ga_2_O_3_ structure.

The deconvoluted O1s spectra in Fig. S4 depict the characteristic of Ga–O at 530.7 eV. The relative intensity of Ga–O deconvoluted peak is reversely proportional to the concentration of oxygen vacancy in the Ga_2_O_3_ films. Figure 4c shows the values of relative proportional component (RPC) of vacancies which are calculated by using Ga–O peak intensities in the RTA Ga_2_O_3_ samples. The improved nitrogen doping at higher annealing temperature is confirmed when the proportional intensity of Ga–O bonds increased at higher RTA temperature. It is also accompanied by the decreased component of oxygen vacancies. On the other hand, the decrease in intensity of Ga3d (Fig. 4d) and Ga2p (Fig. S5) confirms the incorporation of N_2_ in the Ga_2_O_3_ structure. It is also accompanied by the decrease in proportional Ga–O bonds intensity. The N1s core-level spectrum at 397 eV (Fig. 4e) arises from the oxygen substitution by N atoms (acceptor atoms) [12, 20], which is the XPS characteristic of the Ga–N bonding [10]. Without nitrogen, the oxygen and Ga form Ga–O bonding. The presence of N_2_ in annealing process facilitates the formation of Ga–N and Ga–N–O bonds. Lower electronegativity of N (3.0) compared with higher electronegativity of O (3.5) leads to the gradual shift of O1s and Ga3d peaks to lower binding energies in XPS spectra (Fig. 4b, d). Considering larger ionicity of Ga–O bonds than that of Ga–N bonds, the decrease in optical bandgap was predicted by incorporation of nitrogen atoms into Ga–O structure [16]. The Ga–N characteristic bonding was also observed in the Raman spectra (Figs. 2b and S6). The lattice distortion is expected by incorporation of nitrogen atoms into Ga–O lattice structure [17, 18]. A larger d-spacing was reported due to doping of Ga_2_O_3_ film by N_2_ atoms which resulted in the narrower bandgap [17, 18].

### Memristor Devices

**Fig. 5:**
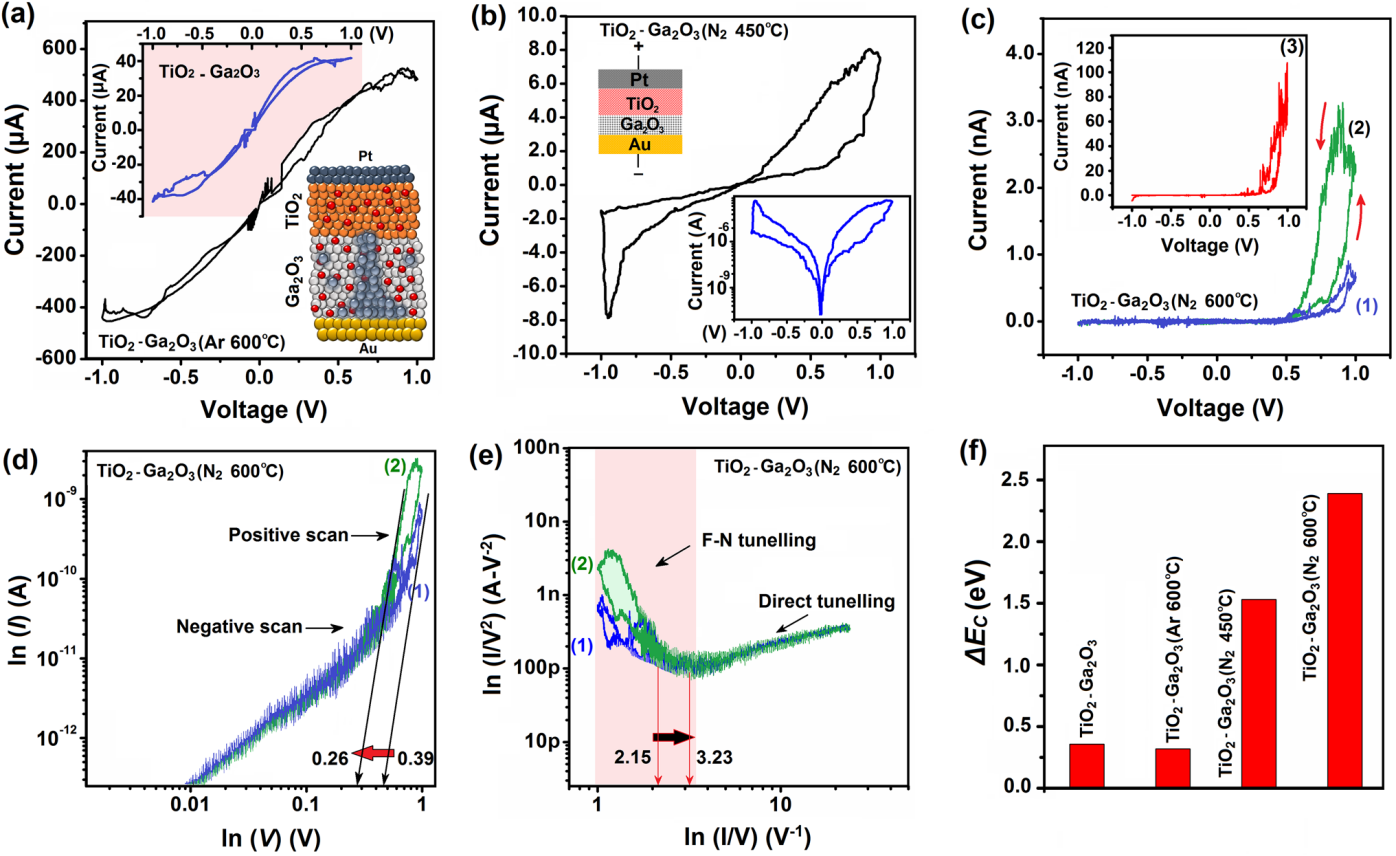
The electrical characterization of fabricated memristor devices accompanied by the characterization of resistive behavior of them. **a** Typical *I*–*V* curves of Pt/TiO_2_–Ga_2_O_3_/Au and Pt/TiO_2_–Ga_2_O_3_ (Ar-600 °C)/Au, **b** Pt/TiO_2_–Ga_2_O_3_ (N_2_-450 °C)/Au, **c** Pt/TiO_2_–Ga_2_O_3_ (N_2_-600 °C)/Au memristor devices for three cycles. **d** Logarithmic scale *I*–*V* characteristics for two cyclic voltammetry of Pt/TiO_2_–Ga_2_O_3_ (N_2_-600 °C)/Au memristor devices. **e** Logarithmic scale of *I/V*^2^ versus the *I/V* curves of the same device. The transition from direct tunneling to F–N tunneling is depicted. **f** Δ*E*_c_ of fabricated heterostructured memristor devices

The control of the charge transfer phenomenon in the memristor devices is the fundamental bases of neuromorphic-based technology [21]. It is expected that the high-bandgap heterostructured films can fulfill the requirement of neuromorphic units since the charge transfer can be tuned and controlled by the manipulation of the heterointerfaces [22]. Furthermore, both transparency and electronegativity differences between the heterostructured components can facilitate the development of bio-inspired optoresponsive instrument. In doing so, 20.0-nm-thick TiO_2_ film was deposited by ALD on as-deposited and RTA Ga_2_O_3_ (5.0 nm) films to fabricate Pt/TiO_2_–Ga_2_O_3_/Au devices. The Raman studies demonstrate the vibration modes of Ga_2_O_3_ and TiO_2_ in heterostructured films (Fig. S7). To understand the charge transfer mechanisms, the current–voltage (*I*–*V*) measurements were performed under dark condition, while the Pt/TiO_2_ was biased and Au/Ga_2_O_3_ was grounded. Typical bipolar switching curves were obtained during the sweeping from 0 → 1→0 → − 1 → 0 for Au/Ga_2_O_3_–TiO_2_/Pt and Au/Ga_2_O_3_(Ar)–TiO_2_/Pt (Fig. 5a). Fragile loop openings were observed in both devices. The driven current from the Au/Ga_2_O_3_(Ar-600 °C)–TiO_2_/Pt sample is tangibly higher than that of as-deposited TiO_2_–Ga_2_O_3_ samples (Fig. 5a). The plausible explanation can be attributed to higher level of Ga excess in RTA (Ar-600 °C) samples compared with that of as-deposited Ga_2_O_3_. It also can be related to the improved crystallinity of Ga_2_O_3_ film after RTA process. The phase-transformed Ga_2_O_3_ with higher level of Ga excess performs as high-conductive layer between TiO_2_ film and Au electrode (Note S1 and Fig. S8). The migration of oxygen vacancies in thicker TiO_2_ layer and charge trapping phenomenon at the heterostructured interface are the most plausible explanations for the resistive behavior of samples [22, 23]. In fact, the thermal annealing of the heterostructured film in oxygen atmosphere has resulted in the deterioration of RS characteristics of them. It can be attributed to the decline of vacancy-related charge trapping sites [24] (Fig. S9). However, still the memristive switching and performance of Ga_2_O_3_–TiO_2_ and Ga_2_O_3_(Ar-600 °C)–TiO_2_ devices are not considerable (Fig. 5a). The heterointerface engineering was employed to alter the charge transfer mechanism between two semiconductor components and to improve the memristive characteristics of heterostructured semiconductor devices. To this aim, ultra-thin Ga_2_O_3_ film was annealed in nitrogen atmosphere to manipulate the heterointerfaces band alignment and also affect the charge trapping and transfer mechanisms at Ga_2_O_3_–TiO_2_ heterointerfaces.

The cyclic *I*–*V* curves for Pt/TiO_2_–Ga_2_O_3_ (N_2_-450 °C)/Au device (Fig. 5b) showed considerable loop opening. During *I*–*V* sweeping, the cell was set to the low-resistance state (LRS) at the positive voltage and reset to the high-resistance state (HRS) at the negative voltage. This process is well known as the *counter*-*eight*-*wise*-*switching* mechanism [25]. A considerable improvement of HRS/LRS ratio was observed after modification of TiO_2_–Ga_2_O_3_ semiconductor heterointerfaces by incorporation of N_2_ atoms into Ga_2_O_3_ film. While the current and resistance values changed gradually for the forward biased, they both changed abruptly at the reverse-biased voltage (logarithmic plot of *I*–*V* in Figs. 5b and S10). The memristive behavior of Pt/TiO_2_–Ga_2_O_3_ (N_2_)/Au device was tangibly different with the performance of TiO_2_/Pt/Ga_2_O_3_ (Ar)/Au samples. Strong rectification behavior (Fig. 5c) was observed during the cyclic *I*–*V* test of TiO_2_–Ga_2_O_3_ (N_2_-600 °C), which is the characteristic of the development of type-II heterojunctions [25]. The loop opening, as characteristic of the charge trapping/de-trapping, is observed again. Comparing the first cyclic loop, it was found that the driven current for TiO_2_–Ga_2_O_3_ (N_2_) was tangibly lower than that for the TiO_2_–Ga_2_O_3_ (Ar). It was also discovered that the driven current from Pt/TiO_2_–Ga_2_O_3_ (N_2_-600 °C)/Au device (Fig. 5c) was tangibly lower than that of the same device annealed at 450 °C (Fig. 5b). Pt/TiO_2_–Ga_2_O_3_ (N_2_-600 °C)/Au heterointerfaces also showed the rectification behavior. It can be understood by investigation of charge trapping mechanisms and determination of energy band alignment at semiconductor heterointerfaces. To realize the underlying dynamics of the charge transfer across the heterostructure, double logarithmic scale *I*–*V* curves of Pt/TiO_2_–Ga_2_O_3_ (N_2_-600 °C)/Au devices are plotted at the several cyclic loops (Fig. 5d). The slope value close to one is the characteristic of the ohmic-like conductance (*I*∝*V*) at lower positive bias voltage, which is caused by the thermally generated free carriers [26]. Trap-filled-limited voltage (*V*_th_) shifted from 0.39 V at the first cycle down to 0.26 V at the second cycle (Fig. 5d). Thus, it is estimated that the trap-filled-limited condition is the main mechanism of the charge trapping at higher voltage [27]. It further confirms the occurrence of charge trapping phenomenon across the 2D heterointerfaces between TiO_2_ and Ga_2_O_3_ (N_2_-600 °C) films. The nonlinear variation of the current at higher voltage is fitted by the Child’s law (*I*∝*V*^2^). The charge trapping phenomena in TiO_2_ film and at the TiO_2_–Ga_2_O_3_ (N_2_) heterointerfaces are expected to be the main mechanism for the resistive behavior at the middle range biased voltages. In the last stage of *I*–*V* curve, which is called the *trap-filled*-*limited* region, an abrupt increase in current is observed when the voltage passes the threshold limit (*V*_TFL_). By imposing higher voltage (*V *>* V*TFL ~ 0.9 V), the device resistance changed from HRS to LRS. The above-mentioned observations confirmed the importance of the trap-controlled space-charge-limited current (SCLC) mechanism [28] in the resistive switching of heterostructured device. The graph of ln (1/*V*^2^) versus the ln (1/*V*) has depicted the transition from the direct to the Fowler–Nordheim (F–N) tunneling [29] by gradual increase in the biased voltage. Graph showed that the specific inflection point was shifted to higher values after the first and second cyclic test (Fig. 5e). While two individual transport regimes were observed in the positive biased voltage, the device only experienced one transport regime corresponding to the negative biased voltages. It was also found that the band alignment of TiO_2_–Ga_2_O_3_ interface could be altered by RTA treatment of the Ga_2_O_3_ component (Figs. S11–S15 and Note S2). It was observed that the Δ*E*_*c*_ value of heterostructured film increased considerably at the TiO_2_–Ga_2_O_3_ (N_2_-600 °C) heterointerfaces. It depicts the tangible difference between the conduction band energy levels of heterostructured component (Figs. 5f and S11). Therefore, it is expected that the heterointerface manipulation and the bandgap adjustment tangibly affected the charge transfer mechanisms in the Ga_2_O_3_–TiO_2_ heterointerfaces. Nitrogen as a strong acceptor can dope and diffuse into Ga_2_O_3_ film and alter the charge carrier mechanism [30]. Different memristive and rectification behavior of TiO_2_–Ga_2_O_3_ (N_2_) devices can be related to different levels of N_2_ incorporation into Ga_2_O_3_ film at two annealing temperatures of 450 and 600 °C (XPS results in Fig. 4e). The Hall-effect measurements clearly demonstrated considerable decrease in the number of charge carriers at the Ga_2_O_3_ thin film annealed in N_2_ atmosphere. It confirms the compensation impact of the nitrogen acceptors on the oxygen-vacancy donors (Fig. S16).

**Fig. 6:**
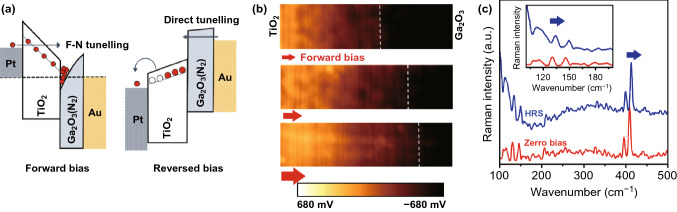
**a** Schematic band diagram for charge transfer from TiO_2_ to Ga_2_O_3_ (N_2_) devices. **b** KPFM analysis monitors the surface potential of memristor device between Au and Pt electrodes for a horizontally developed TiO_2_–Ga_2_O_3_ heterostructure. The scanning area was 1 μm^2^. The bright area represents the electron trap regions, and the dark area depicts the hole trap regions. **c** Raman spectra of TiO_2_ film of memristor device in zero bias and HRS mode

A trapping-induced bipolar RS model is elaborated to explain the nonvolatile charge trapping mechanisms in the Pt/TiO_2_–Ga_2_O_3_ (N_2_)/Au devices. It can be explained by considering the type of band alignment (type II) and the driving internal electric field caused by the potential difference between the Ga_2_O_3_ and TiO_2_ heterointerfaces components. Taking into account the bias polarity on TiO_2_ and Ga_2_O_3_ samples, the charge trapping/de-trapping process can happen either in semiconductor component or at the heterointerfaces. Considering the noble nature of Au and Pt electrodes, the electrochemical oxidation and reduction of electrodes are not expected to affect the resistive switching mechanisms. The trap level of oxygen vacancy in the TiO_2_ film was estimated to be ~ 0.7 eV below the conduction band edge [31], and the calculated Schottky barrier height at the Pt/TiO_2_ films is 0.7 eV (Note S3 and Fig. S17). Under the forward-biased voltage, the empty trap sites in the TiO_2_ film keep the device in HRS [32]. On the other hand, by applying efficient voltage on the Pt electrode, the energy level of the trap sites is pulled down until it reached the level below the Fermi energy of the Pt electrode. Consequently, the electrons could be injected into TiO_2_ layer either by direct tunneling or by F–N tunneling through the Pt–TiO_2_ Schlocky junction at higher biased voltage. The injected electrons then gradually fill the trapping sites (Fig. 6a). When the trapping sites are filled by electrons completely, the unique band alignment in the TiO_2_–Ga_2_O_3_ heterointerfaces can still suppress and trap the other injected electrons at the TiO_2_–Ga_2_O_3_ interface (Fig. 6a). As the biased voltage increased, the electrons pass the heterointerfaces barrier and flow through the ultra-thin RTA-treated Ga_2_O_3_ film. Here, the annealing process facilitated the charge transfer through the Ga_2_O_3_ layer since it can increase the amount of the metallic Ga excess in the Ga_2_O_3_ barrier layer and facilitate the HRS to LRS transition. By removing the forward-biased voltage, the band alignment in heterointerfaces returns to its initial condition. Thus, the instant de-trapping of charges occurs by imposing the reverse-biased voltage. Due to the internal electric field in the TiO_2_–Ga_2_O_3_, the traps stay longer and decaying process takes more time to be completed. The noisy characteristics of *I*–*V* curves in Fig. 5c can be attributed to the charge de-trapping [32]. These charges are expected to be trapped at higher energy levels compared with the Fermi level of the Pt electrode. The trapping-induced bipolar RS mechanism was previously observed in the heterostructured all-oxide-based resistive memories in neuromorphic devices [32–35]. Therefore, to monitor the potential across the heterointerfaces and to investigate migration of the oxygen vacancies, a specific horizontal configuration of the TiO_2_–Ga_2_O_3_ device is designed. This configuration of heterostructured oxide facilitates the overhead monitoring of samples by the Kelvin probe force microscopy (KPFM) (Fig. S18). To this aim, several snapshots of the surface potential of samples were monitored, while the forward potential was increasing (Fig. 6b). The brighter parts in the images are the manifestation of electron-trapped regions in which the surface potential is higher than that of the non-trapped parts [36]. The gradual increasing area of the bright region reinforces the proposed mechanism for the charge trapping and resistive switching at the heterointerfaces [37]. A uniform potential drop across the region between two heterointerfaces components was observed. It confirms the gradual filling of the trapping sites in the main oxide component of heterostructured film, i.e., TiO_2_ film. To further investigate the effect of oxygen vacancies on the resistance of memristor, the Raman measurements were carried out to monitor the TiO_2_ film at HRS and zero-bias conditions (Fig. 6c). The Raman characterization of heterointerfaces at the zero-biased state and at the HRS displayed the red shift of O1s and Ti–O Raman characteristic peaks after charge trapping phenomenon (Fig. 6c). It was observed that the occupation of trapping sites is accompanied by the decrease in concentration of the oxygen vacancies. This observation confirms the effect of oxygen-vacancy sites on the charge trapping mechanism.

### Nociceptor Devices

**Fig. 7:**
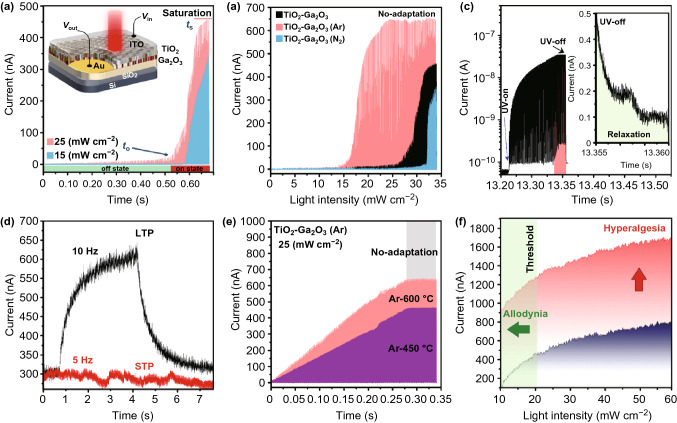
The photoinduced nociceptive behavior of devices. **a** The light intensity-dependent photoresponse of ITO/TiO_2_–Ga_2_O_3_/Au devices under 0.1 V bias showing the threshold and saturation characteristics. **b** Comparison of the typical photoresponse of various memristor devices showing the effect of heterointerface engineering on threshold and saturation characteristics of fabricated memristor devices. The ITO/TiO_2_–Ga_2_O_3_ (N_2_-600 °C)/Au nociceptive device shows the highest threshold values among various heterostructured devices. **c** Relaxation characteristics of ITO/TiO_2_–Ga_2_O_3_ (N_2_-600 °C)/Au nociceptive device under 35 mW cm^−2^ pulsed laser (360 nm) illumination. **d** The effect of light frequency on relaxation time of nociceptor devices. **e** Allodynia characteristics of ITO/TiO_2_–Ga_2_O_3_ (Ar)/Au devices. **f** Allodynia and hyperalgesia of ITO/TiO_2_–Ga_2_O_3_ (Ar)/Au devices

The trapping/de-trapping characteristics of our all-oxide-heterostructured nociceptor were accompanied by the internal electric field caused by the difference in electron affinity of TiO_2_ and Ga_2_O_3_ films. These observations bring the idea of the development of optically modulated resistive neuromorphic device. The fabricated samples are highly transparent (~ 80%) with the optical bandgap in the UV region (Fig. S19). This instrument is quite similar to the eye’s cornea which is also highly transparent and has the highest number of nociceptors. Inset in Fig. 7a depicts a typical unit structure in which the transparent indium tin oxide (ITO) layer is deposited over Au/Ga_2_O_3_–TiO_2_ heterostructure to make a conductive transparent electrode. Then, the device photoresponse was measured by imposing 0.1 V bias voltage under illumination of a tunable UV laser (360 nm). By increasing the light intensity to 25 mW cm^−2^ the photoresponse across the Au/Ga_2_O_3_–TiO_2_/ITO device reached toward the saturation (Fig. 7a). This phenomenon is essential prerequisite parameters to satisfy the nociceptive characteristics of device. It was observed that the decrease in light intensity was accompanied by the delay of photoresponse initiation time and the delay of saturation time (*t*_s_) of optical nociceptors (Fig. 7a). In ITO/TiO_2_–Ga_2_O_3_ (Ar-600 °C)/Au device, the photoresponse appeared and increased suddenly when the magnitude of light intensity was higher than 15 mW cm^−2^ (Fig. 7b). This observation indicates that the tangible photoinduced charge transport happens when enough photoinduced carriers are generated by the pulsed light. This is the basis of the threshold nature of fabricated optical nociceptor device. The magnified view of photocurrent response of nociceptor before and after the threshold is presented in Fig. S20. The distinguished nociceptor photoresponsivity in on- and off-states confirms that the threshold phenomenon has quite significant impact on nociceptive performance of fabricated devices. As an incredible observation, it was found that the required light intensity to turn on and then to get the saturation state in TiO_2_–Ga_2_O_3_ (N_2_-600 °C) device is higher than that of TiO_2_–Ga_2_O_3_ and TiO_2_–Ga_2_O_3_ (Ar-600 °C) devices (Fig. 7b). Furthermore, it was observed that the *t*_0_ and *t*_s_ *of* TiO_2_–Ga_2_O_3_ unit are higher than those of TiO_2_–Ga_2_O_3_ (Ar-600 °C) device. It is observed that the turn-on time and saturation time of TiO_2_–Ga_2_O_3_ (N_2_-600 °C) device are the highest value among all nociceptors. TiO_2_ is considered as the main charge generation and trapping component of all-oxide transparent heterostructure. Considering the similarity of TiO_2_ thickness in all devices, the *t*_0_ and *t*_s_ values are mostly attributed to the charge transfer phenomenon in heterointerfaces. It was discovered that the *ΔE*_c_ of TiO_2_–Ga_2_O_3_ (Ar-600 °C) heterointerfaces is the smallest among all instruments, while the *ΔE*_c_ of TiO_2_–Ga_2_O_3_ (N_2_-600 °C) heterostructured film is the highest one. This higher barrier height in heterointerfaces can explain why it takes longer time and needs higher energy for TiO_2_–Ga_2_O_3_ (N_2_) device to turn on and then to reach to the saturation state. These distinctions among our heterostructured devices enabled us to fabricate either high-sensitive TiO_2_–Ga_2_O_3_ (Ar) or high-threshold TiO_2_–Ga_2_O_3_ (N_2_) nociceptors. It was found that after saturation, the device photocurrent does not change even by increasing the power density of the pulsed light (Fig. 7b). This behavior is similar to the *no*-*adaptation* characteristics of the natural human nociceptors, which protects the organs from the unnecessary and harmful external stimuli [38]. The signal relaxation is another important characteristic of the fabricated nociceptors. It explicitly refers to the required time for the ultimate decay of the response current after the elimination of light stimuli. Figure 7c depicts that the current increased suddenly and stayed constant for the rest of stimulation at 13.3 s. Our observations confirmed that the relaxation time is dependent on the stimuli frequency where the increased light frequency has resulted in longer relaxation time (Fig. 7d). The device property is similar to the human nociceptors when a stronger pain is caused by intense and continuous stimuli and the pain lasts longer until it completely disappears. Furthermore, at higher frequencies the response signals overlap, resulting in the nociceptor charge accumulation (Fig. 7d). In this state, the remained generated photo-carriers of the previous spike facilitate the device conductance in the following stimulation stage. Similar behavior was observed in the synaptic units, which is called excitatory postsynaptic current (EPC). The EPC corresponds to the synaptic weight of a biological synapse which can be transferred from the short-term plasticity (STP) to the long-term potentiation (LTP) mode [39]. This characteristic is also similar to the human nociceptors when even a weak hazardous stimulus triggers a strong chronic pain.

Figure 7e shows the photoresponsivity of ITO/TiO_2_–Ga_2_O_3_ (Ar)/Au units where *allodynia* characteristic is observed in the damaged states. As it is depicted, at the constant light intensity of 25 mW cm^−2^ the threshold times (*t*_o_) have shifted to the initial illumination stage. It was demonstrated the RTA samples (Ar-600 °C) provided higher photoresponsivity. The nociceptor behaved similar to a receptor at this state. However, since the light intensity is constant (25 mW cm^−2^), the *no*-*adaptation* state is still observed. Interestingly, the TiO_2_–Ga_2_O_3_ (N_2_) devices do not show the *allodynia* at the same light stimulation (Fig. S21), which vividly depicts the heterointerfaces manipulation effect on nociceptive behavior. In addition, when the light intensity is increased, the *hyperalgesia* phenomenon is also detected in TiO_2_–Ga_2_O_3_(Ar) nociceptors (Fig. 7f). The long-term stability of nociceptors was confirmed after conducting several tests on other devices with the same configuration and preparation steps. Figure S22 demonstrates the cyclic *I*–*V* curves of ITO/TiO_2_–Ga_2_O_3_ (N_2_)/Au device at several cyclic sequences and under the illumination of light source with different intensities. The memristive behavior of heterostructured semiconductor device under light illumination was different compared with the performance of device in darkness. The combined effects of increased light intensity and higher cyclic number made the device to face its photocurrent threshold where the no-adaptation state occured. The photogenerated electron and holes evidently affected the trapping and transfer of charge carriers in all-oxide heterostructured device, as it was reported previously in the study of resistive behavior of heterostructured oxide semiconductor devices [40]. On the basis of experimental observations, a plausible model has been introduced to explain the operating mechanism of our optoelectronic nociceptor (Fig. S23). The tangible difference between the electron affinity of TiO_2_ and Ga_2_O_3_ accompanied by the imposed biased voltage facilitates the transfer of the photogenerated charges from TiO_2_ to Ga_2_O_3_. The type-II heterointerfaces cause charge trapping at the positive biased voltage. Because of the interfacial barrier height, the electrons mostly transport from TiO_2_ to Ga_2_O_3_ film by F–N tunneling mechanism [41]. The developed heterointerfaces can act as the rapping site for generated electrons and holes after removing the light stimuli source. The N_2_-doped Ga_2_O_3_ acts as the hole acceptor component of heterostructure. Under the impact of the applied electric field, the generated holes drift toward the N_2_-doped Ga_2_O_3_. If the light stimulus is strong enough, the generated charge carriers remain highly stable at the heterointerfaces and increase the relaxation time.

## Conclusions

All-oxide-heterostructured optoresponsive nociceptor with controllable charge transfer is fabricated. Newly designed nociceptor utilized ultra-thin sub-stoichiometric amorphous TiO_2_–Ga_2_O_3_ heterostructures. Ultra-thin Ga_2_O_3_ films were thermally annealed in Ar and N_2_ atmospheres. It was discovered that the phase reconfiguration in Ga_2_O_3_ was accompanied by the substantial jump in its conductivity induced by the thermally assisted redox reaction of amorphous nanostructure in Ar atmosphere. The artificial nociceptor has clearly demonstrated the threshold, relaxation, *allodynia*, and *hyperalgesia* states closely resembling the human bio-nociceptor behavior. The artificial nociceptor functions are based on unique switching behavior and outstanding dynamics of memristor. It was experimentally confirmed that the charge transfer can be tuned and controlled by the interfaces manipulation in the ultra-thin heterostructures. The heterointerface manipulation ultimately enabled fabrication of either high-sensitive TiO_2_–Ga_2_O_3_ (Ar) nociceptor or the high-threshold TiO_2_–Ga_2_O_3_ (N_2_) nociceptor. The newly designed optoresponsive nociceptor can be readily readjusted to be responsive to other stimuli such as chemicals. Such versatility can have profound impact on wide range of applications where the human presence is considered as extremely hazardous. Moreover, due to its bio-realistic capabilities and scalability advantage over the existing microstructured counterparts, developed nociceptors are highly desirable to be utilized in micro- and nano-robotics at various environmental conditions.

## Electronic supplementary material

Below is the link to the electronic supplementary material.

Supplementary material 1 (PDF 2192 kb)
