# Supplementary material for: Nanoscale All-Oxide-Heterostructured Bio-inspired Optoresponsive Nociceptor
Source: Nanomicro Lett. 2020 Apr 1;12:83. doi: 10.1007/s40820-020-00419-z (PMC7770938; doi:10.1007/s40820-020-00419-z)
Supplement: Supplementary file 1 — Supplementary material 1 (PDF 2192 kb) [file 40820_2020_419_MOESM1_ESM.pdf]

Supporting Information for

Nanoscale All-oxide-heterostructured Bio-inspired Optoresponsive Nociceptor

Mohammad Karbalaei Akbari[1, 2,*], Jie Hu[3], Francis Verpoort[4], Hongliang Lu[5], Serge Zhuiykov[1, 2, *]

[1]Centre for Environmental & Energy Research, Ghent University Global Campus, Incheon, South Korea

[2]Department of Green Chemistry & Technology, Faculty of Bioscience Engineering, Ghent University, 9000 Ghent, Belgium

[3]College of Information Engineering, Taiyuan University of Technology, Taiyuan, Shanxi 030024, People's Republic of China

[4]Laboratory of Organometallics, Catalysis and Ordered Materials, State Key Laboratory of Advanced Technology for Materials Synthesis and Processing, Wuhan University of Technology, Wuhan 430070, People's Republic of China

[5]School of Microelectronic, Fudan University, Shanghai 200433, People's Republic of China

*Corresponding authors. E-mail: Serge.Zhuiykov@ugent.be (Serge Zhuiykov); Mohammad.akbari@ugent.be (Mohammad Karbalaei Akbari)

Supplementary Figures

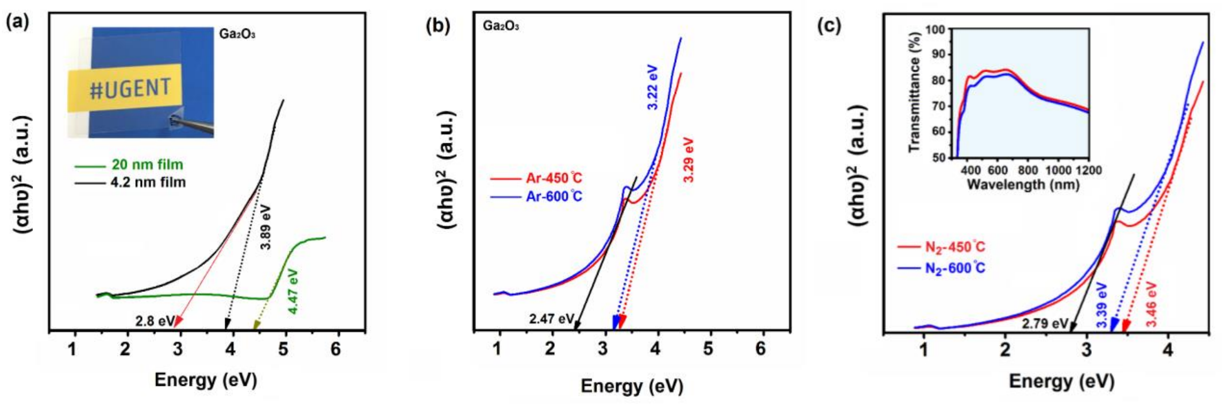

Fig. S1 The Bandgap measurement of samples. (**a**) The bandgap of as-deposited 20 and 5 nm gallium oxide. Inset shows highly transparent gallium oxide films over ITO substrate. (**b**) The bandgap of gallium oxide films after RTA and quenching in Ar atmosphere at different annealing temperatures. (**c**) The bandgap measurements of gallium oxide films after RTA and quenching in N_2 atmosphere

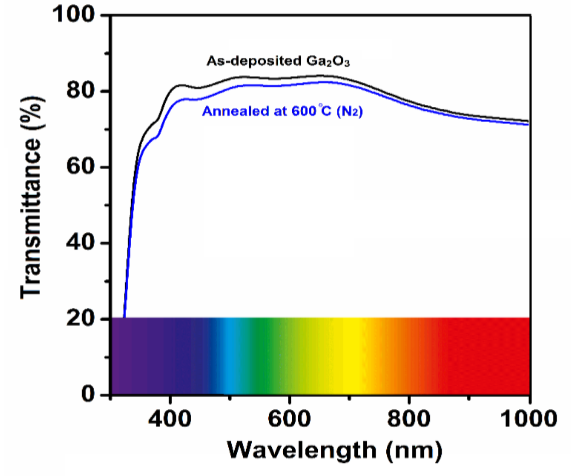

Fig. S2 Transmittance spectra of as-deposited gallium oxide and RTA gallium oxide sample in N_2 atmosphere

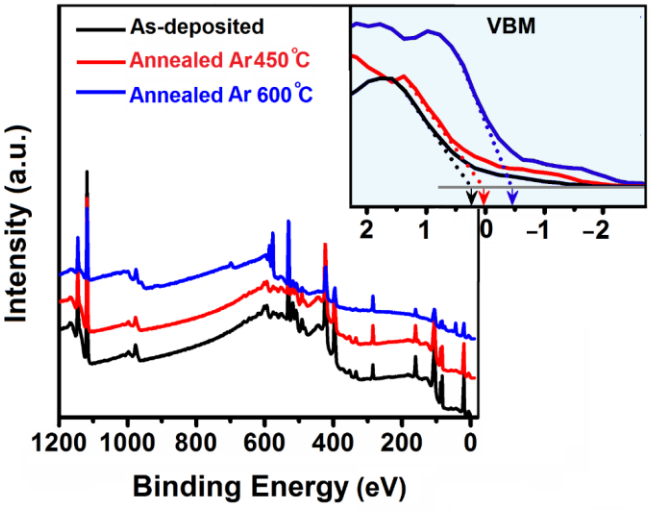

Fig. S3 Changes of valence band maximum (VBM) in as-deposited gallium oxide and RTA gallium oxide sample in Ar atmosphere

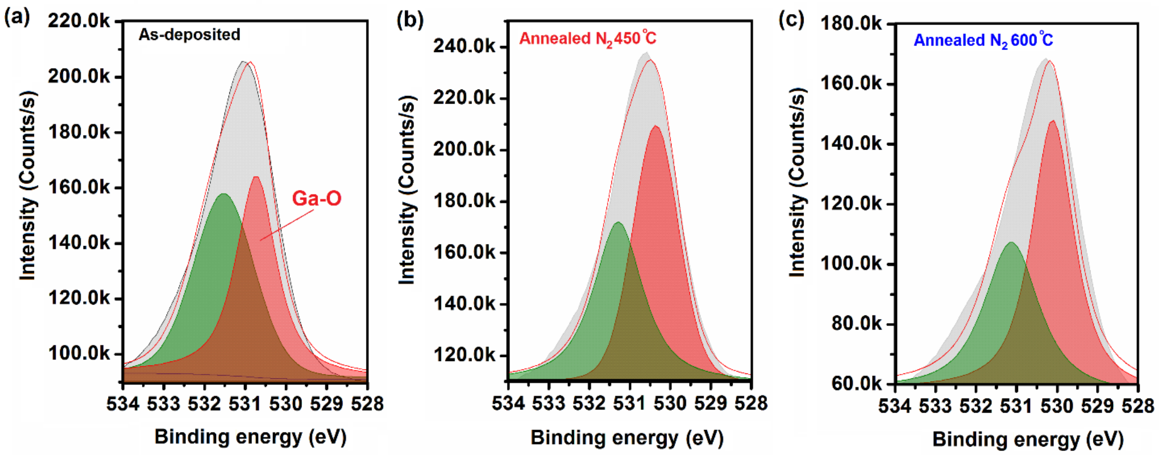

Fig. S4 O 1S XPS spectra of (**a**) as-deposited and RTA (N_2) samples at (**b**) 450 °C and (**c**) 600 °C

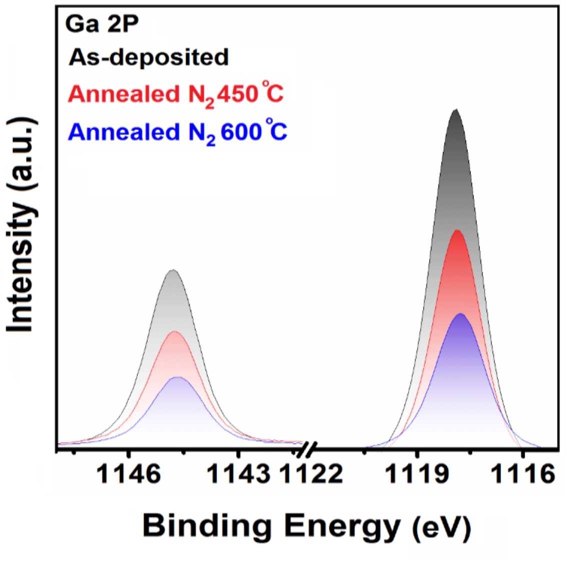

Fig. S5 Ga 2P XPS spectra of as-deposited and RTA (N_2) samples at 450 and 600 °C

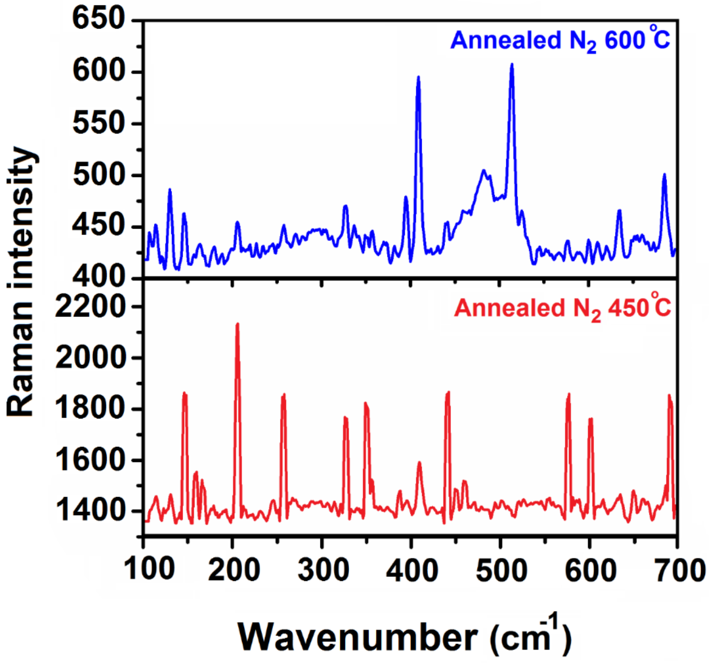

Fig. S6 The Raman spectra of RTA (N_2) samples at 450 and 600 °C. Graph shows the decreased intensity of Ga-O attributed bonding and increased intensity of Ga-N bonding

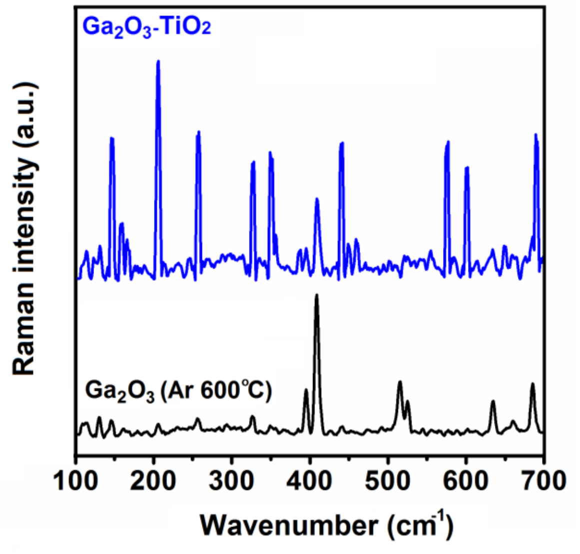

Fig. S7 Raman spectra of TiO_2-Ga_2O_3 RTA sample in Ar. Graph shows the decreased intensity of Ga-O attributed bonding and increased intensity of Ga-N bonding

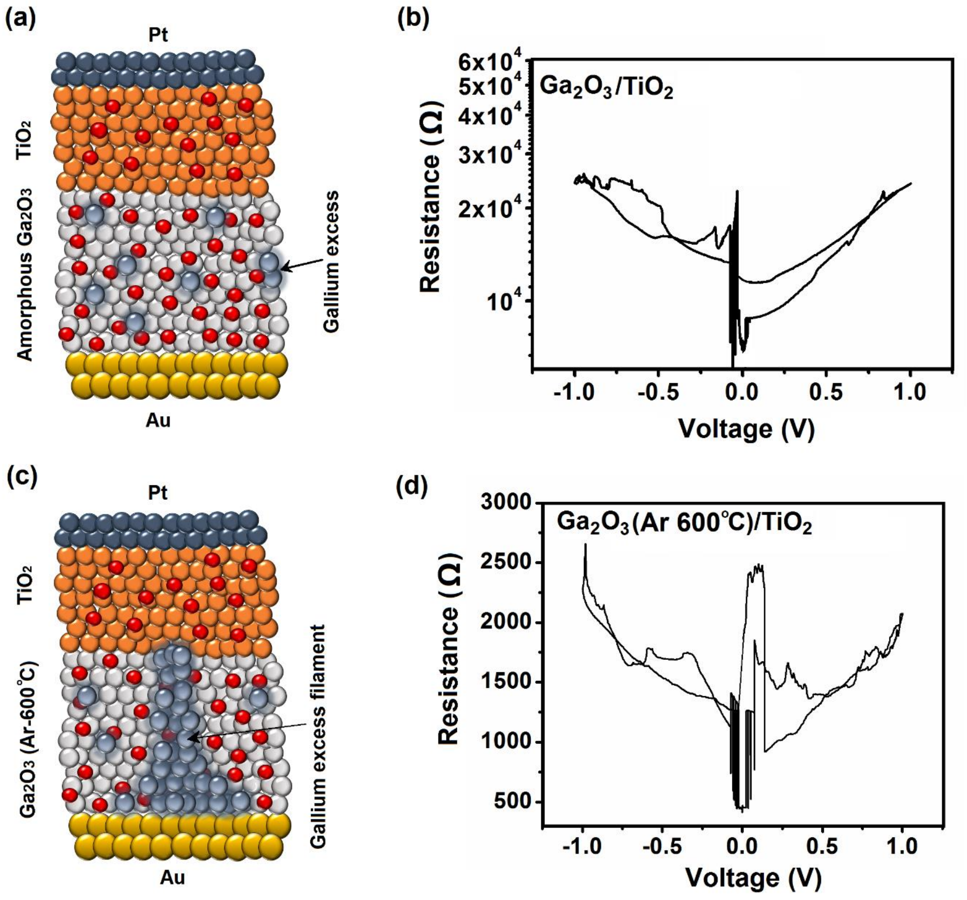

Fig. S8 (**a**) The graphical scheme of Pt/TiO_2-Ga_2O_3/Au device accompanied by the (**b**) typical resistance variation of the same device in cyclic *I-V* curve. (**c**) The graphical scheme of Pt/TiO_2-Ga_2O_3 (Ar-600S °C)/Au device accompanied by the (**d**) the typical resistance variation of the same device in cyclic *I-V* curves

Supplementary Note S1 The amorphous gallium oxide contains Ga excess. The amount of Ga excess is increased in the microstructure which is due to the nucleation of crystalline Ga_2O_3 due to Ga rejection into remained amorphous structure. By increasing the Ga excess, the creation of a conductive channel of metallic gallium is facilitated which plays the role of a conductive bridge between Au electrode and TiO_2 film. Consequently the resistance of device decreased tangible as it can be compared from the Fig. S8b, d.

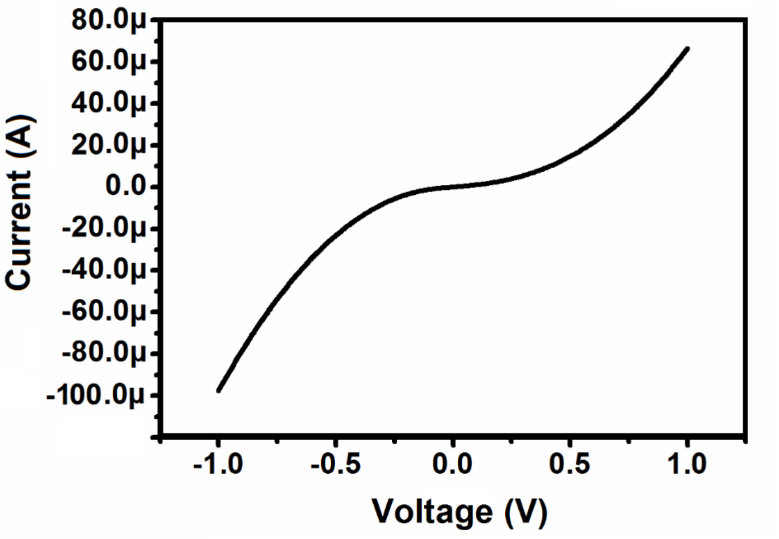

Fig. S9 Typical logarithmic *I-V* curves of Pt/TiO_2- Ga_2O_3/Au device after annealing in oxygen atmosphere

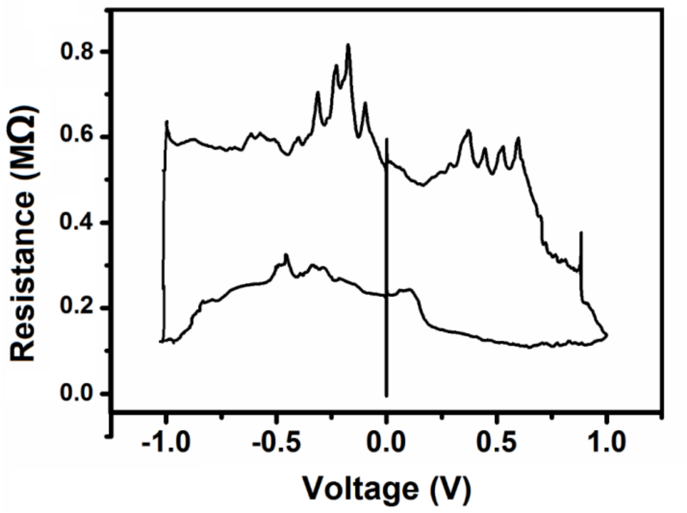

Fig. S10 The resistance variation in Pt/TiO_2-Ga_2O_3 (N_2-450 °C)/Au device in cyclic *I-V* measurements

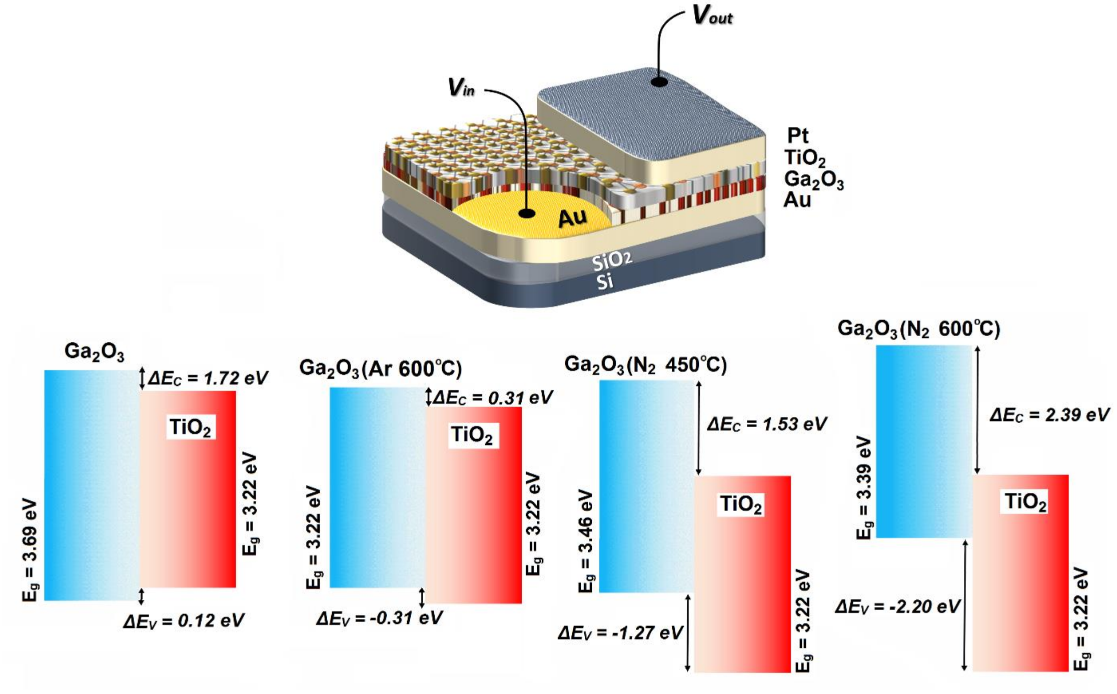

Fig. S11 Band alignment in TiO_2-Ga_2O_3 heterostructured devices

Supplementary Note S2 Band Alignment Calculation in TiO_2-Ga_2O_3 Heterostructure

Based on Kraut's method, the valence band offset (VBO) can be extracted by Eq. S1 [S1, S2]:

$$\Delta E_V = \left(E_{Ga\ 2P}^{Ga_2O_3} - E_{VBM}^{Ga_2O_3}\right) - \left(E_{Ti\ 2P}^{TiO_2} - E_{VBM}^{TiO_2}\right) - \left(E_{Ga\ 2P}^{Ga_2O_3} - E_{Ti\ 2P}^{TiO_2}\right) \qquad (S1)$$

In which $E_{Ga\ 2P}^{Ga_2O_3}$ is core level (CL) spectra of Ga 2p, $E_{VBM}^{Ga_2O_3}$ is the valence band maximum (VBM) of Ga_2O_3, $E_{Ti\ 2P}^{TiO_2}$ is the CL of Ti 2P spectra, $E_{VBM}^{TiO_2}$ is the VBM of TiO_2. To calculate the VBM of Ga_2O_3 and TiO_2, the XPS spectra of TiO_2-Ga_2O_3 were used (Figs. S12-S15). To describe the integrated band offsets of TiO_2-Ga_2O_3 (Ag) heterojunction, the corresponding energy difference between conduction bands can be calculated from Eq. S2 [S1, S2]:

$$\Delta E_C = E_{Bandgap}^{Ga_2O_3} - E_{Bandgap}^{TiO_2} - \Delta E_V \qquad (S2)$$

Using the above equation and by employment of the XPS spectra of Ga_2O_3, TiO_2, and TiO_2-Ga_2O_3 (Ag) heterostructure, the band alignment diagram of TiO_2-Ga_2O_3 heterostructures can be calculated. The values of ΔE_V and ΔE_C for the TiO_2-Ga_2O_3 (Ag) heterostructure are -1.95 and 3.57 eV, respectively. Based on bandgap measurements and band alignment calculation, the simplified band diagrams of TiO_2-Ga_2O_3 heterostructures are shown in Fig. S11, which show Type II heterointerfaces.

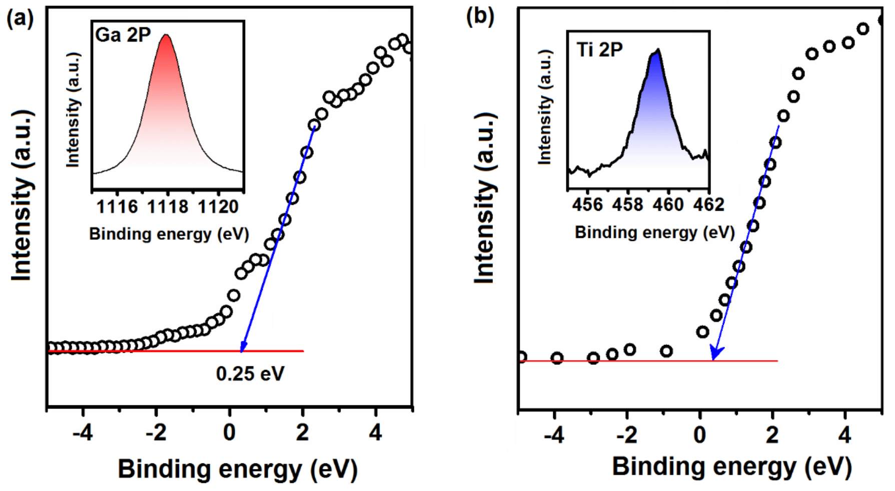

Fig. S12 VBM of the Ga_2O_3 and TiO_2 films by extrapolating XPS graphs

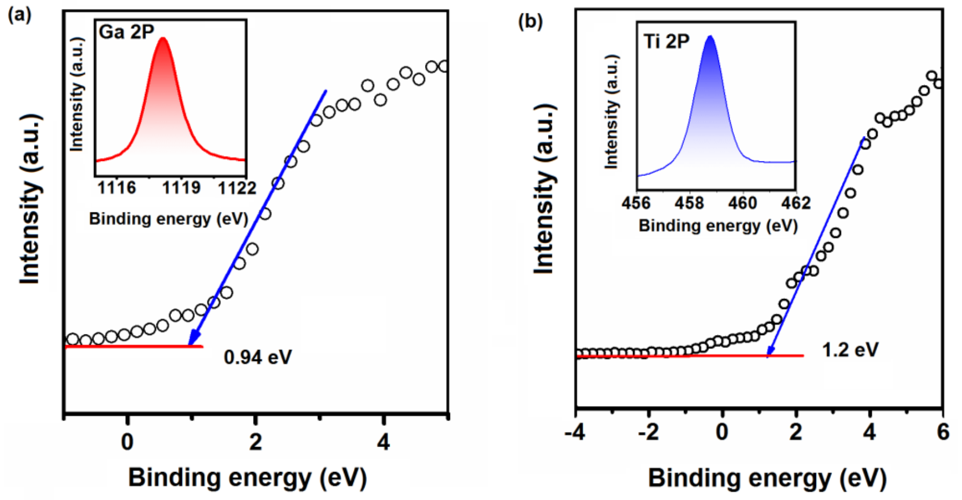

Fig. S13 VBM of the Ga_2O_3 (Ar-600 °C) and TiO_2 films by extrapolating XPS graphs

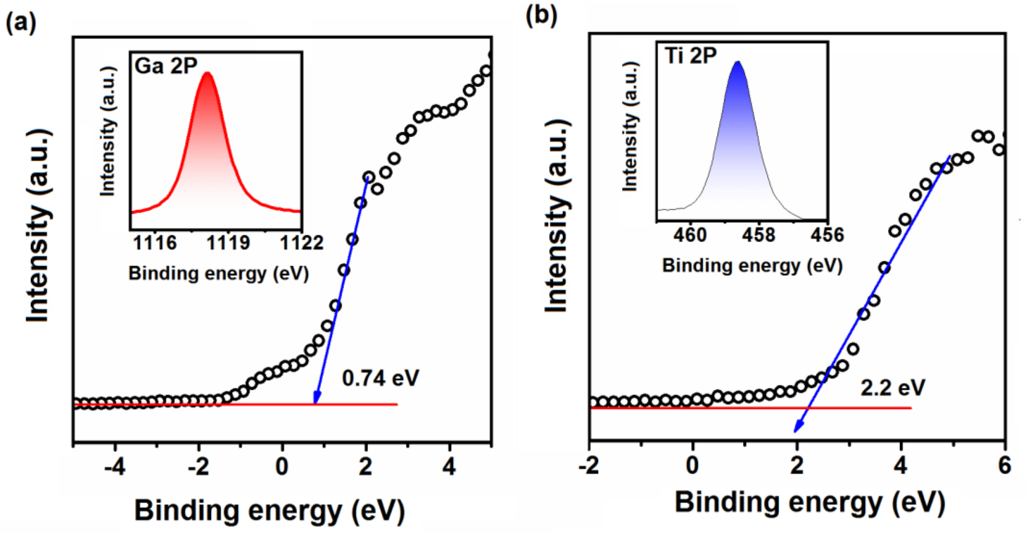

Fig. S14 VBM of the Ga_2O_3 (N_2-450 °C) and TiO_2 films by extrapolating XPS graphs

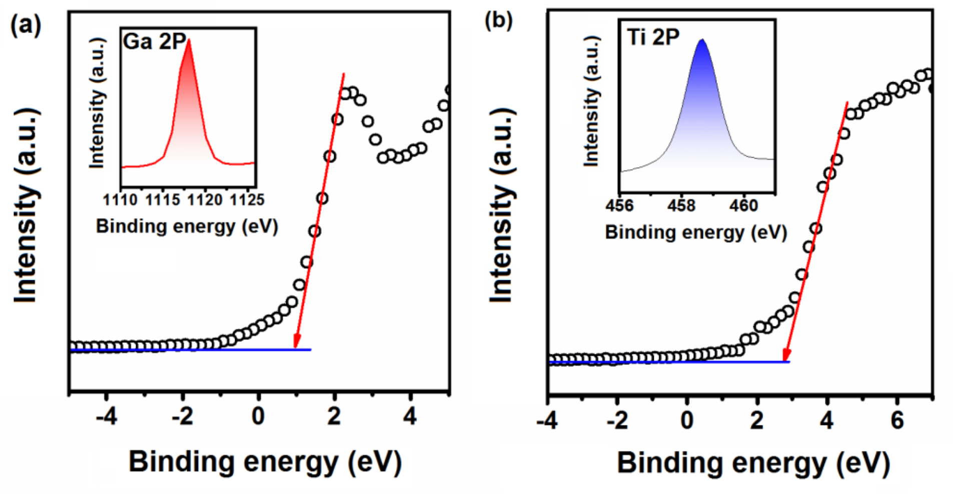

Fig. S15 VBM of the Ga_2O_3 (N_2-600 °C) and TiO_2 films by extrapolating XPS graphs

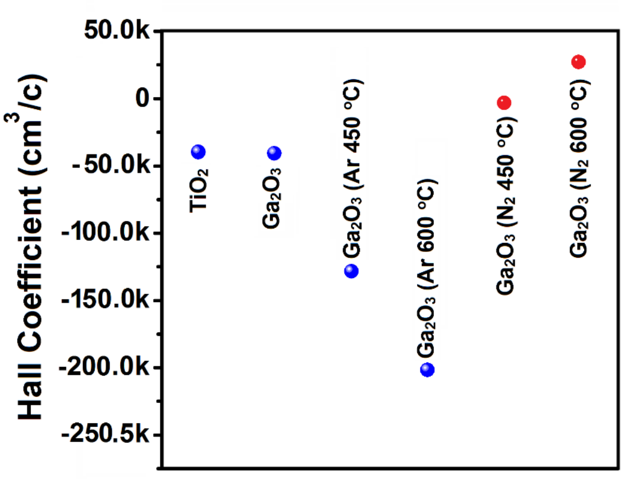

Fig. S16 The results of Hall-effect measurements of heterostructured films

Supplementary Note S3-Calculation of Schottky Barrier Height

Barrier height of a metal–semiconductor contact can be experimentally measured and determined by using I-V curves. Considering that the current is due to thermionic emission, the relation between the applied forward bias and current can be expressed by Eq. (S3) [S3]:

$$J = J_0 \exp\left(\frac{qv}{nkt}\right) \qquad \text{(S3)}$$

Where *n* is ideally factor, T is the temperature in Kelvin, *q* is the electron charge, *k* is the Boltzmann constant and I_0 is the reverse saturation current which can be extracted by extrapolation the straight line of lnI to intercept the axis at zero voltage. The Schottky barrier height (SBH) can be calculated by extrapolation of semi-logarithmic *J-V* curves to V=0. The SBH can be calculated from Eqs. (S4) and (S5):

$$\emptyset_B = \frac{kT}{q} \mathrm{Ln} \frac{T^2 A^*}{J_0} \qquad \text{(S4)}$$

$$A^* = \frac{4\pi m^* k^2}{h^3} \qquad \text{(S5)}$$

Where m^* is the effective electron mass, and h is the Planck's constant. The A^* is the effective Richardson constant[4,5] which is equal to 41.1 A cm^{-2} K^{-2}. The experimental *J-V* characteristics and the logarithmic scale of the same graphs are shown in Fig. S17. We used low forward bias of *J-V* curves to measure the SBH at Pt/TiO_2 junctions. Taking the logarithmic version of Eq. S3, we can extract the n and I_0 from the slop and Y axis of *LnJ-V* plot. After performing least square fitting on the *LnJ-V* plot in the linear region, the values of n and J_0 from the slop and the Y-axis can be determined.

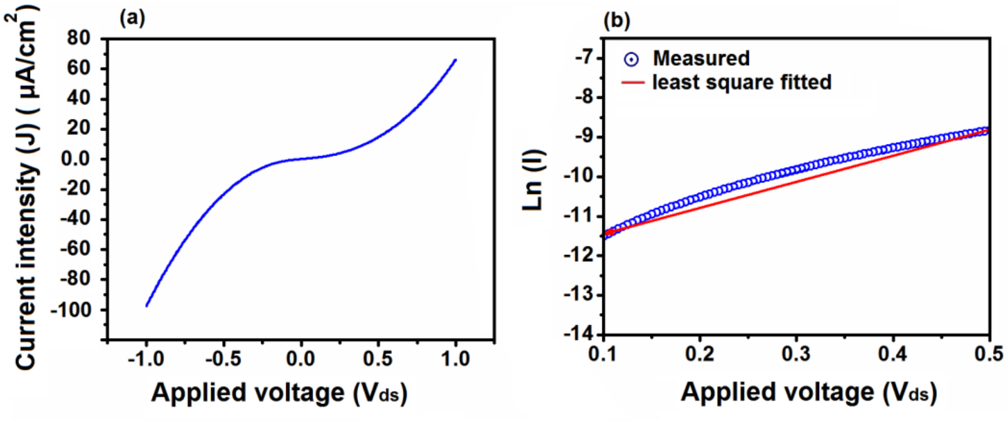

Fig. S17 a *J-V_{ds}* characteristics of Pt/TiO_2 device and **b** *LnJ-V* plot of the Pt/TiO_2 junction (Schottky diode) at the 273 K

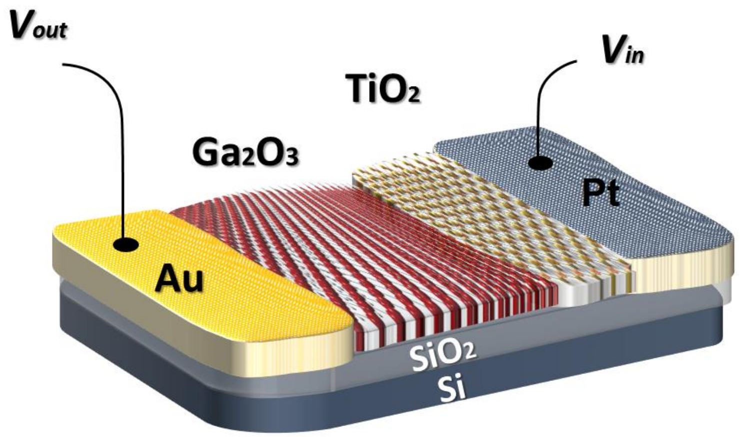

Fig. S18 Graphical scheme of memristor device for KPFM studies and Raman measurements of memristor behavior. The measurements were performed on TiO_2 film

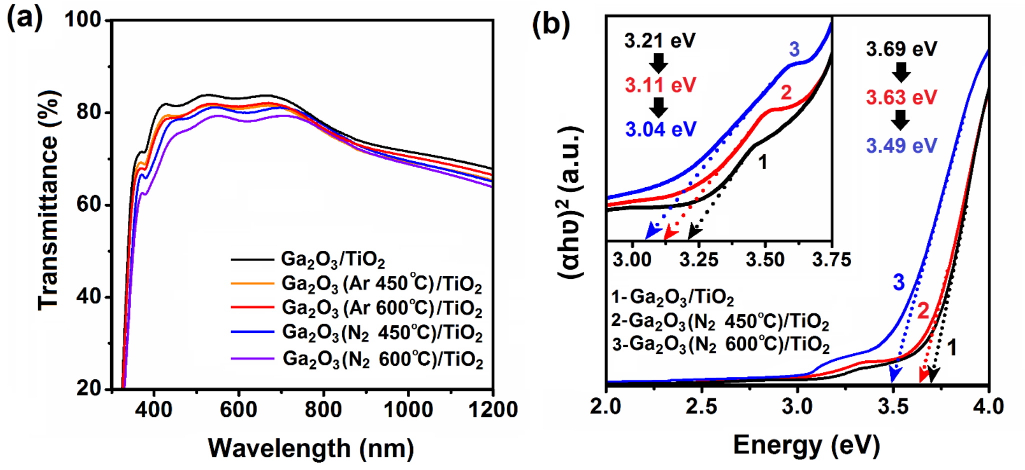

Fig. S19 Transmittance and bandgap of heterostructured TiO_2-Ga_2O_3 devices

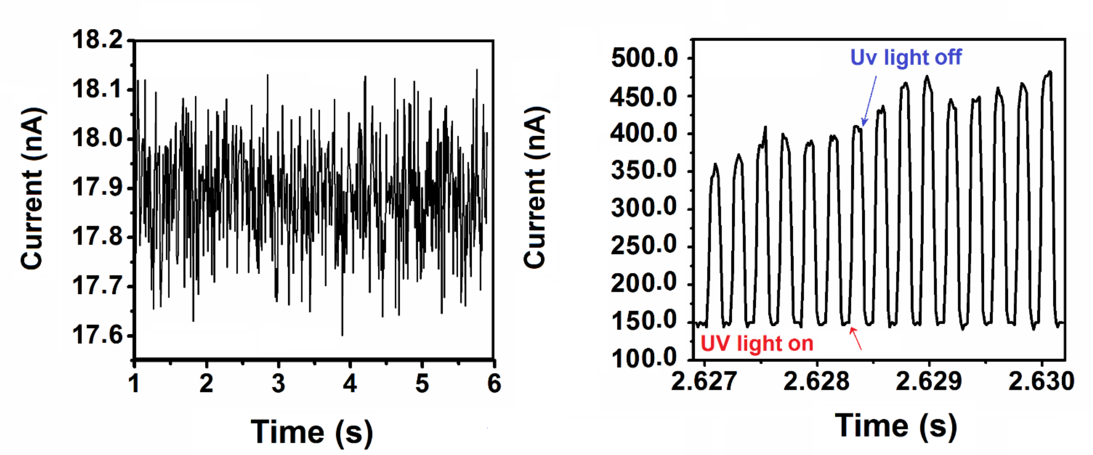

Fig. S20 Photoresponse of heterostructured ITO/TiO_2-Ga_2O_3 (Ar- 600oC)/Au devices before and after threshold of light intensity

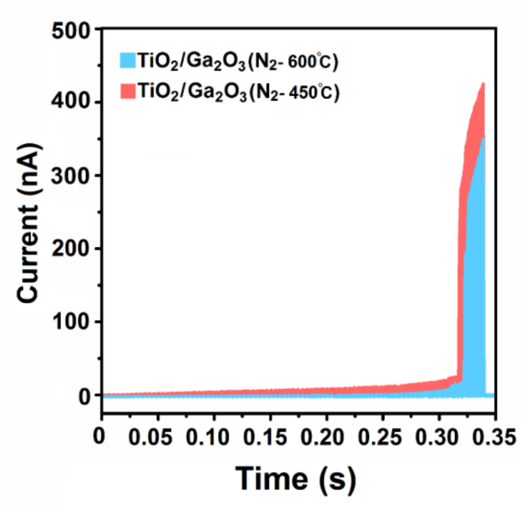

Fig. S21 Photoresponse of heterostructured ITO/TiO_2-Ga_2O_3(N_2)/Au devices at constant light intensity of 25 mW cm^{-2}

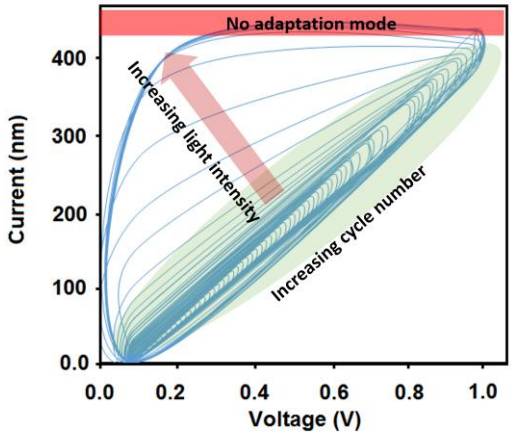

Fig. S22 Photoresponse of heterostructured ITO/TiO_2-Ga_2O_3/Au devices at different cyclic courses and under illumination of different light intensities

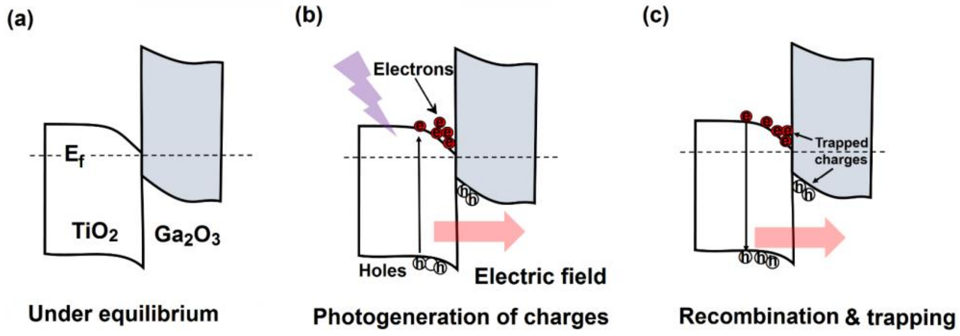

Fig. S23 Proposed mechanism for optically generated charge transfer in TiO_2-Ga_2O_3 heterointerfaces. (**a**) Under equilibrium, (**b**) charge photo generation under electric field and (**c**) photogenerated charge trapping and recombination under electric field. The photogenerated electrons are trapped at TiO_2-Ga_2O_3 heterointerfaces under forward bias voltage. The increase of light intensity is accompanied by the generation of higher charge carriers. Thus, higher number of photogenerated charges are expected to be trapped at TiO_2-Ga_2O_3 heterointerfaces.

Supplementary References

[S1] S.M. Sun, W.J. Liu, Y.P. Wang, Y.W. Huan, Q. Ma et al., Band alignment of In_2O_3/β-Ga_2O_3 interface determined by X-ray photoelectron spectroscopy. Appl. Phys. Lett. **113**, 031603 (2018). https://doi.org/10.1063/1.5038615

[S2] E.A. Kraut, R.W. Grant, J.R. Waldrop, S.P. Kowalczyk, Semiconductor core-level to valence-band maximum binding-energy differences: Precise determination by x-ray photoelectron spectroscopy. Phys. Rev. B **28**, 1965 (1983). https://doi.org/10.1103/PhysRevB.28.1965

[S3] S. Gholami, M. Khakbaz, Measurement of I-V characteristics of a PtSi/p-Si Schottky barrier diode at low temperatures. WASET **57**, 1001-1004 (2011).

[S4] L.A.M. Lyle, *Gallium Oxide Technology, Device and Applications*, 1st edn. (EDs: S. Pearton, F. Ren, M. Mastro, Elsevier Science, 2019).

[S5] K. Sasaki, M. Higashiwaki, A. Kuramata, T, Masui, S. Yamakoshi, Ga_2O_3 Schottky barrier diodes fabricated by using single-crystal β-Ga_2O_3 (010) substrates. IEEE Electron Device Lett. **34**, 493 (2013). https://doi.org/10.1109/LED.2013.2244057
